# Inhibition of 11β-Hydroxysteroid Dehydrogenase Type II Suppresses Lung Carcinogenesis by Blocking Tumor COX-2 Expression as Well as the ERK and mTOR Signaling Pathways

**DOI:** 10.1371/journal.pone.0127030

**Published:** 2015-05-26

**Authors:** Jian Chang, Min Xue, Shilin Yang, Bing Yao, Bixiang Zhang, Xiaoping Chen, Ambra Pozzi, Ming-Zhi Zhang

**Affiliations:** 1 Department of Medicine, Vanderbilt University School of Medicine, Nashville, TN, United States of America; 2 Department of Cancer Biology, Vanderbilt University School of Medicine, Nashville, TN, United States of America; 3 Hepatic Surgery Center, Tongji Hospital, Tongji Medical College, Huazhong University of Science & Technology, Wuhan, China; 4 Jiangsu Center for the Collaboration and Innovation of Cancer Biotherapy, Cancer Institute, Xuzhou Medical College, Xuzhou, China; University of Parma, ITALY

## Abstract

Lung cancer is by far the leading cause of cancer death. Early diagnosis and prevention remain the best approach to reduce the overall morbidity and mortality. Experimental and clinical evidence have shown that cyclooxygenase-2 (COX-2) derived prostaglandin E_2_ (PGE2) contributes to lung tumorigenesis. COX-2 inhibitors suppress the development and progression of lung cancer. However, increased cardiovascular risks of COX-2 inhibitors limit their use in chemoprevention of lung cancers. Glucocorticoids are endogenous and potent COX-2 inhibitors, and their local actions are down-regulated by 11β–hydroxysteroid dehydrogenase type II (11ßHSD2)-mediated metabolism. We found that 11βHSD2 expression was increased in human lung cancers and experimental lung tumors. Inhibition of 11βHSD2 activity enhanced glucocorticoid-mediated COX-2 inhibition in human lung carcinoma cells. Furthermore, 11βHSD2 inhibition suppressed lung tumor growth and invasion in association with increased tissue active glucocorticoid levels, decreased COX-2 expression, inhibition of ERK and mTOR signaling pathways, increased tumor endoplasmic reticulum stress as well as increased lifespan. Therefore, 11βHSD2 inhibition represents a novel approach for lung cancer chemoprevention and therapy by increasing tumor glucocorticoid activity, which in turn selectively blocks local COX-2 activity and/or inhibits the ERK and mTOR signaling pathways.

## Introduction

Lung cancer is the second most common cancer in both men and women and is by far the leading cause of cancer death among both men and women. The American Cancer Society estimates that about 230 000 new cases of lung cancer will be diagnosed along with approximate 160 000 deaths from lung cancer, accounting for about 27% of all cancer deaths in 2013 in the United States. Most patients present with advanced, non-curable disease. There are only 15% of patients still alive 5 years after diagnosis [1,2]. Therefore, early diagnosis and prevention remain the best approach to reduce the overall morbidity and mortality of lung cancer. There are two major types of lung cancer: small cell lung cancer (SCLC) and non-small cell lung cancer (NSCLC). NSCLC accounts for 85%-90% of lung cancers and contains three main subtypes: squamous cell (epidermoid) carcinoma, adenocarcinoma and large cell (undifferentiated) carcinoma.

Although the etiology of lung cancer is undoubtedly multifactorial, there is experimental and clinical evidence linking abnormalities in the cyclooxygenase/prostaglandin system to its pathogenesis. Cyclooxygenase (prostaglandin synthase G_2_/H_2_, COX) is the rate-limiting enzyme in the metabolism of arachidonic acid to prostaglandin G_2_ and subsequently to prostaglandin H_2_ (PGH_2_), which serves as the precursor for prostaglandin E synthetase to produce prostaglandins [3]. Two isoforms of cyclooxygenase exist in mammals, “constitutive” COX-1 and inflammatory-mediated and glucocorticoid-sensitive COX-2. COX-2 derived PGE_2_ has been reported to promote tumor growth and metastasis through stimulation of cell proliferation, cell migration, cell invasion, angiogenesis and immunosuppression [4].

An increase in COX-2 expression has been associated with the development of different human NSCLC and possibly with acquisition of an invasive and metastatic phenotype, as well as with poor prognosis [5–7]. Notably, a single nucleotide polymorphism in the COX-2 promoter region, a change of -1195 G to A (-1195 G/A SNP) that leads to increases in enzymatic activity, is associated with poor survival and poor progression-free survival in unresectable locally advanced NSCLC [8]. In a randomized, double-blind, placebo-controlled trial, the selective COX-2 inhibitor celecoxib was found to be a potential chemoprevention agent in former-smokers [9]. COX-2 inhibitors have been reported as radiosensitizers for NSCLC patients [10]. However, long-term use of selective COX-2 inhibitors has been found to be associated with an increased incidence of cardiovascular events, thought to be due to inhibition of endothelial cell-derived COX-2 activity, with selective inhibition of COX-2 derived PGI_2_ production but without inhibition of COX-1 mediated prothrombotic platelet thromboxane A_2_ production [11–13].

COX-2 was initially described as an inflammatory-mediated and glucocorticoid-sensitive cyclooxygenase. Glucocorticoids (GCs) are the most potent, endogenous, specific COX-2 inhibitors, acting to suppress COX-2 expression through stimulating glucocorticoid receptors [14–16]. In addition to inhibiting COX-2 expression, GCs also reduce prostaglandin production through inhibition of cytosolic phospholipase A_2_ activity, which prevents the release of arachidonic acid from membrane phospholipids, and through inhibition of microsomal prostaglandin E synthetase (mPGES-1) expression, a major terminal synthetase in PGE_2_ biosynthesis [17,18]. In addition to their application in the treatment of hematologic malignancies, GCs inhibit solid tumor growth, regress tumor mass, and prevent metastasis by blocking angiogenesis [19,20]. However, the undesirable side effects of immune suppression limit their application in cancer chemoprevention and chemotherapy.

The actions of GCs in tissues are modulated by a “pre-receptor” regulatory mechanism involving 11ß–hydroxysteroid dehydrogenase type I (11ßHSD1) and 11ßHSD2 [21]. 11ßHSD1 produces active GCs from inactive metabolites, while 11ßHSD2 converts GCs to their inactive keto-forms. Inhibition of 11ßHSD2 activity increases COX-2 inhibition [22,23]. In the current study, we investigated the expression of 11ßHSD2 in lung cancers and whether inhibition of 11ßHSD2 activity could suppress lung tumorigenesis due to increased tumor cell intracellular active glucocorticoids and subsequent inhibition of COX-2 expression/activity.

## Materials and Methods

### Ethics Statement

All animal experiments were performed according to animal care guidelines and were approved by the Vanderbilt Institutional Animal Care and Use Committe (IACUC) (M/11/130).

### Animals

KrasLA2 mice were a gift of Dr. T. Jacks, MIT [24]. Since the *KrasLA* allele is non-functional in the germline configuration, only heterozygous mice were maintained and used in the experiments. Age- and sex-matched KrasLA2 mice were treated with water (control) or the 11βHSD2 inhibitor, glyccyrrhetinic acid (GA, 10 mg/kg/day, i.p.) from 6 to 20 weeks of age and sacrificed at 20 weeks of age. Under anesthesia with Nembutal (60 mg/kg i.p.), the lungs were weighed, filled with fixative overnight, transferred to 70% ethanol for 24 h, and examined using a dissecting microscope to count surface polyps in a blinded fashion [22]. The tumor diameter on the surface of the lung was measured with a digital caliper. Tumor volume was expressed in mm^3^ and calculated as [*l x w* ^*2*^] × 0.5, where *l* and *w* denote length and width, respectively [22]. After tumors were counted, lung tissues were processed for paraffin embedding and immunohistochemical analysis. A subset of mice was sacrificed for collection of adenomas and lung tissue for immunoblotting and determination of tissue levels of corticosterone and 11-keto-corticosterone. All animals were genotyped twice (after weaning and before sacrifice).

### Survival analysis

KrasLA2 mice were treated with water or 11βHSD2 inhibitor, GA at 6 weeks of age. The animals were monitored daily. The animal was sacrificed at the first sign of shortness of breath, reduced locomotion and reduced body weight (greater than 20% total body weight). Lungs from these mice were analyzed for the presence of visible tumors on the lung surface. Only mice with visible lung tumors were used for the survival analysis. The animals were euthanized by overdose carbon dioxide.

### Human lung cancer tissues

Slides of lung cancer tissue array with normal lung tissue were purchased from US Biomax (Rockville, MD).

### Cell culture

Lewis lung carcinoma (LLC) cells, H2073, H1435, H25, A549, H1944, H1792, H1793, H1299, and H1395 cells were grown in RPMI1640 supplemented with 4,500 mg/l glucose, 2 mM l-glutamine, 10% fetal bovine serum, 100 U/ml penicillin, and 100 μg/ml streptomycin in 5% CO2 and 95% air at 37°C [25]. All cell lines were obtained from American Type Culture Collection (ATCC, Manassas, VA, USA). LLC cells or H1435 cells were cultured in medium with 1% fetal bovine serum overnight and then treated with corticosterone (CS) with or without 11βHSD2 inhibitors, GA (10 μM) or carbenoxolone (CBX, 10 μM), for 8 hours and cells were harvested for COX-2 immunoblotting. The cells were seeded in a 96-well plate (5 x 10^3^ cells in 200 μl medium) for 16 hours, then starved for 24 hours in medium containing 0.1% fetal bovine serum, and various concentrations of corticosterone (CS) or CS plus 10μM GA were added for additional 4 hours. Then BrdU solution was added to each well for 4 hours. Analysis was performed according to the manufacturer’s protocol (BrdU cell proliferation assay kit, Cat#6813, Cell Signaling) and our recent report [26].

### Primary tumor growth

For mouse LLC tumor experiments, male C57/B6 mice (8–12 weeks old) were given 2 dorsal s.c. injections (5 x 10^5^ cells in 100 μl PBS) with administration of water (control, i.p) or GA (10 mg/kg/day, i.p.) begun 1 day prior to cell injection. Tumors were harvested and weighed after 18 days of growth. In another set of experiment, LLC cell suspension containing 5 x 10^5^ cells in 100 μl PBS were injected into tail vein with or without GA administration as described above. The time of death, or when mice became moribund (by the same criteria as the endpoints described for survival studies), was recorded for survival analysis.

### Measurement of corticosterone and 11-keto-corticosterone

Lung tissues from KrasLA2 mice were collected and stored at-80°C. Tissue levels of corticosterone (active form in rodent) and 11-keto-corticosterone (inactive form) were measured using high-performance liquid chromatography coupled with electrospray tandem mass spectrometry [22]. Corticosterone was from ICN Biomedicals, and 11-keto-corticosterone was from Steraloids.

### Antibodies

Affinity-purified rabbit anti-11βHSD2 (catalog no. BHSD22-A) was purchased from Alpha Diagnostic International; rabbit anti-murine COX-2 (catalog no. 160106) was from Cayman Chemicals; rabbit anti-CHOP (catalog no. 2895), rabbit anti-cleaved caspase-3 (catalog no. 9661), rabbit anti-p-ERK (T202/Tyr204, catalog no. 4370), p-mTOR (Ser2448, catalog no 2976), p-PERK (Thr980, Catalog no 3179) and p-eIF2α (Ser51, catalog no 9721) were from Cell Signaling; rabbit anti-cyclin D1 (catalog no. SC-753) was from Santa Cruz Biotechnology; mouse anti-mannose receptor (MR, CD206, catalog no.MAB25341) was from R&D; rabbit anti-Ki67 (ab15580) was from Abcam.

### Immunohistochemical staining and immunoblotting

Immunostaining was carried out as in previous reports [27]. For immunostaining of phosphorylated proteins, antigen retrieval was achieved by boiling in citric acid buffer (100 mM, pH 6.0) for 3 x 5 min. Lung tumors isolated from 20 weeks old KrasLA2 mice were used for immunoblotting as described previously [28].

### Quantitative image analysis

Immunostaining was quantified by using the BIOQUANT image analysis system (R & M Biometrics, Nashville, TN) [22]. Bright-field images from a Leitz Orthoplan microscope with DVC digital RGB video camera were digitized and saved as computer files. Contrast and color level adjustment (Adobe Photoshop) were performed for the entire image; i.e., no region- or object-specific editing or enhancements were performed.

### Statistical analysis

Values are presented as means ± S.E.M. ANOVA and Bonferroni 2-tailed *t*-test were used for statistical analysis, and differences were considered significant for *P* values **<** 0.05.

## Results

**Fig 1:**
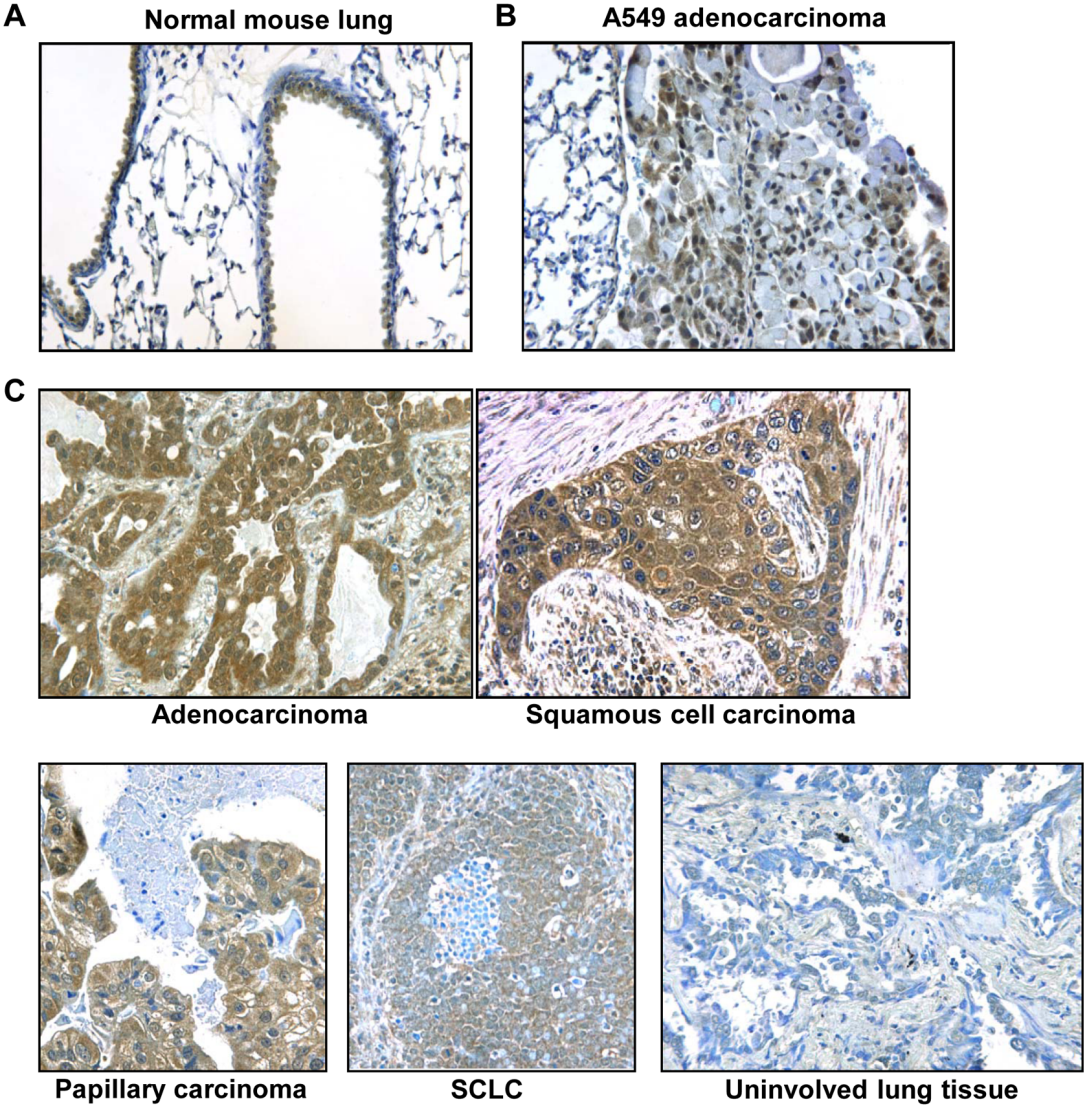
11ßHSD2 expression in mouse and human lung tumors. **A** and **B**. Representative photomicrographs indicated 11ßHSD2 expression in small airway and alveolar epithelial cells of athymic nude mice **(A)** and in A549 lung tumor from athymic nude mice **(B)**. **C**. Representative photomicrographs showed that 11ßHSD2 immunostaining was strong in lung adenocarcinoma and squamous cell carcinoma, moderate in papillary carcinoma and small cell lung cancer (SCLC), but very weak in uninvolved lung tissue. Original magnification: x 160 in all.

To investigate whether 11ßHSD2 might be involved in lung tumorigenesis, its expression in normal mouse lung tissue, experimental lung cancer, normal human lung tissue and different human lung cancers was investigated with immunohistochemistry. In athymic nude mouse lung tissue, 11ßHSD2 was expressed in the epithelial cells of small airways and alveoli (Fig 1A). 11ßHSD2 expression was increased in A549 adenocarcinoma compared to adjacent normal athymic nude mouse lung tissue (Fig 1B). 11ßHSD2 immunostaining was strong in human adenocarcinoma and squamous cell carcinoma, moderate in papillary carcinoma as well as small cell lung cancer, but very weak in normal human lung tissue (Fig 1C). Negative control was performed by replacing the primary antibody with purified rabbit IgG and no immunoreactivity was found (data not shown). Therefore, 11ßHSD2 expression is increased in both experimental lung cancers and human lung cancers.

**Fig 2:**
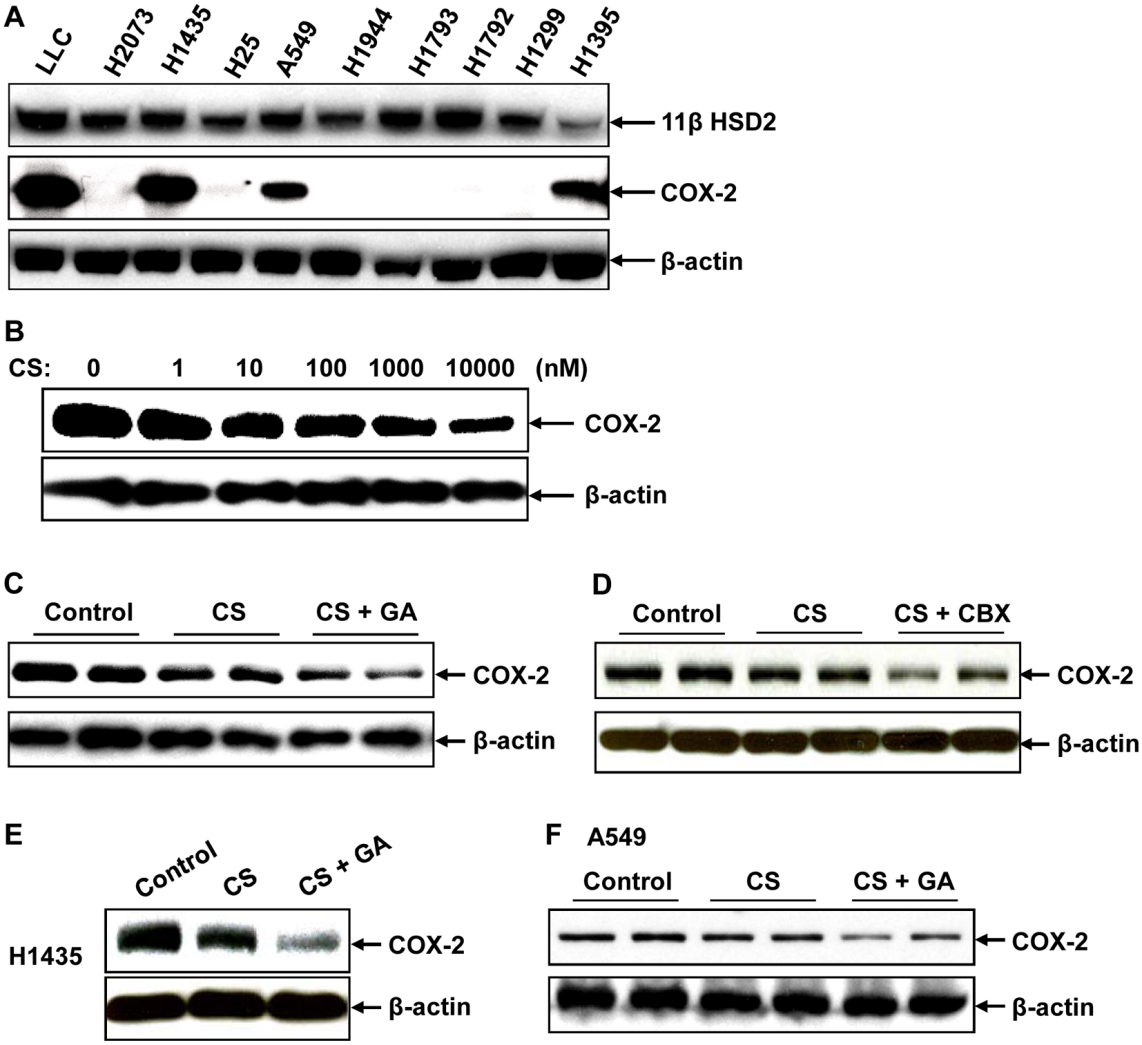
11βHSD2 inhibition augmented corticosterone (CS)-induced COX-2 inhibition in lung cancer cells. **A**. Immunoblotting indicated that 11βHSD2 was expressed in all investigated cell lines while COX-2 was detectable in some of them, including LLC, H1435 and A549 cells. **B**. Immunoblotting indicated that CS inhibited LLC cell COX-2 expression in a dose-dependent manner. **C**. CS (1μM)-induced LLC cell COX-2 inhibition was enhanced by 11βHSD2 inhibitor, GA. **D**. CS (1μM)-induced LLC cell COX-2 inhibition was enhanced by carbenoxolone (CBX, 10 μM), another 11βHSD2 inhibitor. **E**. CS (1μM)-induced H1435 cell COX-2 inhibition was enhanced by GA (10 μM). **F**. CS (1μM)-induced A549 cell COX-2 inhibition was enhanced by GA (10 μM).

To investigate whether inhibition of 11ßHSD2 activity was beneficial in lung tumorigenesis through inhibition of COX-2 expression due to increased intracellular active glucocorticoids, we screened lung cancer cell lines for 11ßHSD2 and COX-2 expression. As indicated in Fig 2A, 11ßHSD2 was expressed in all investigated lung cancer cell lines; while COX-2 was only detected in LLC, H1435, A549 and H1395 cells. LLC cell line, which is highly tumorigenic in mice and expresses both COX-2 and 11ßHSD2, was chosen to investigate the role of 11ßHSD2 inhibition in COX-2 expression. As indicated in Fig 2B, corticosterone (CS, glucocorticoid in rodent) inhibited LLC cell COX-2 expression in a dose-dependent pattern. However, even 1μM CS could not completely inhibited COX-2 expression. One possible reason for this partial inhibition is that intracellular CS is quickly inactivated by 11ßHSD2. It is also possible that some COX-2 is insensitive to glucocorticoid inhibition. CS-induced LLC cell COX-2 inhibition was markedly enhanced in the presence of GA, an inhibitor of 11ßHSD2 activity (Fig 2C). In addition, CS-induced LLC COX-2 inhibition was also enhanced by carbenoxolone (CBX, 10 μM), another inhibitor of 11ßHSD2 activity (Fig 2D) [29]. Furthermore, CS-induced COX-2 inhibition was also enhanced by GA treatment in both H1435 and A549 cells, which also express both 11ßHSD2 and COX-2 (Fig 2E and 2F).

**Fig 3:**
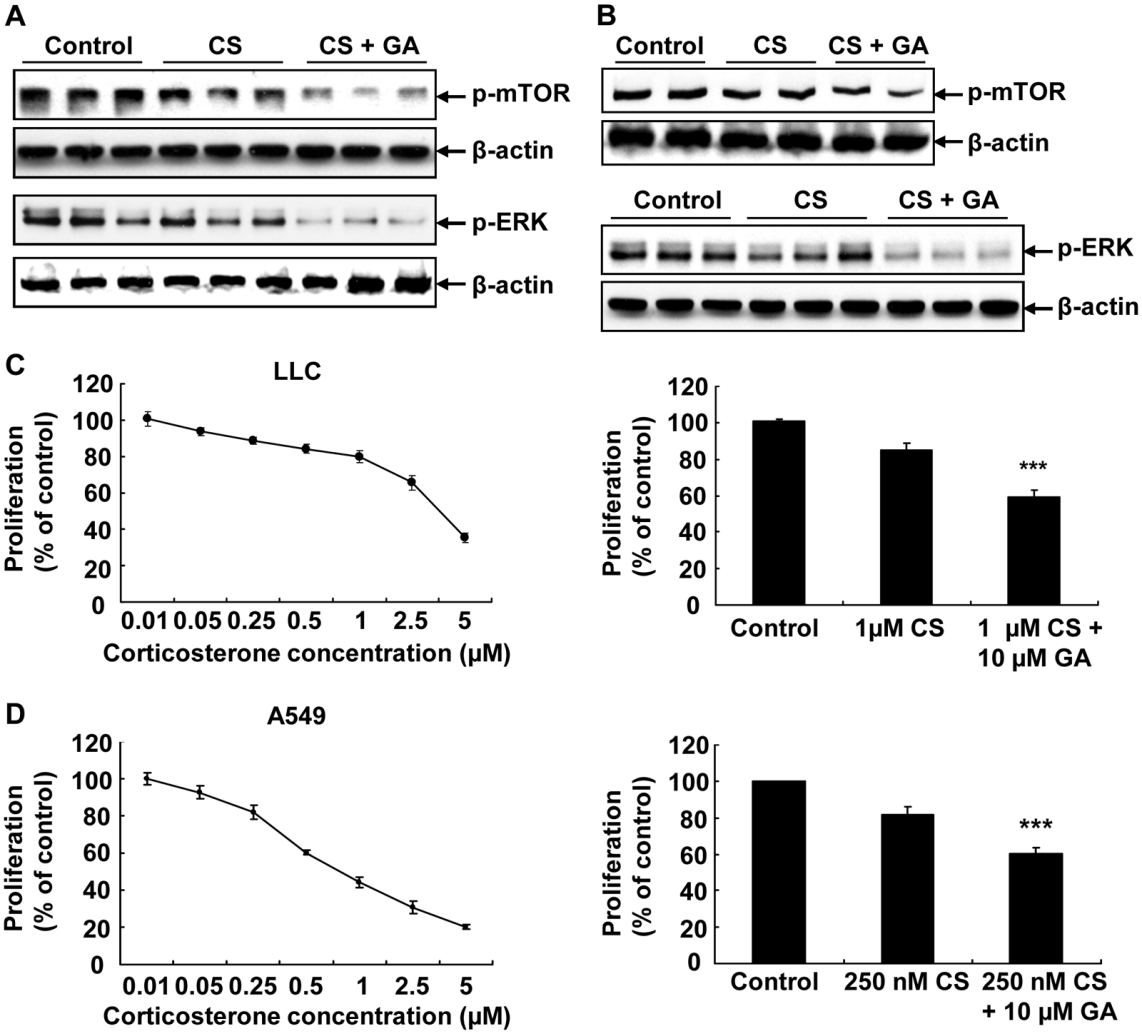
CS-induced inhibition of mTOR and ERK signaling pathways and cell proliferation was enhanced by 11βHSD2 inhibition with GA in lung cancer cell lines. **(A and B)**. CS treatment led to decreased expression levels of p-mTOR and p-ERK in LLC cells **(A)** and A549 cells **(B)**, which were augmented by GA treatment. **(C and D)**. CS treatment led to inhibition of LLC **(C)** and A549 **(D)** cell proliferation in a dose-dependent manner, which was enhanced by GA treatment. ***P < 0.001, n = 3.

To determine the effect of 11ßHSD2 inhibition on CS-mediated inhibition of mTOR and ERK signaling pathways, both LLC cells and A549 cells were treated with 1 μM CS with or without 10 μM GA for 24 hours and p-mTOR and p-ERK levels were determined with immunoblotting. As indicated in Fig 3A, CS treatment resulted in decreases in both p-mTOR and p-ERK levels in LLC cells, which were enhanced by 11ßHSD2 inhibition with GA. Similarly, CS-mediated inhibition of p-mTOR and p-ERK was also augmented by GA treatment in A549 cells (Fig 3B).

To investigate the direct effects of CS on lung cancer cell proliferation, we treated LLC cells (mouse) and A549 cells (human) with different dose of CS and cell proliferation was measured by using cell proliferation assay kit. As shown in Fig 3C, CS inhibited LLC cell proliferation in a dose-dependent pattern. In addition, CS-mediated inhibition was significantly enhanced by addition of GA. Similarly, CS-mediated inhibition of A549 cell proliferation was also augmented by GA treatment in A549 cells (Fig 3D).

**Fig 4:**
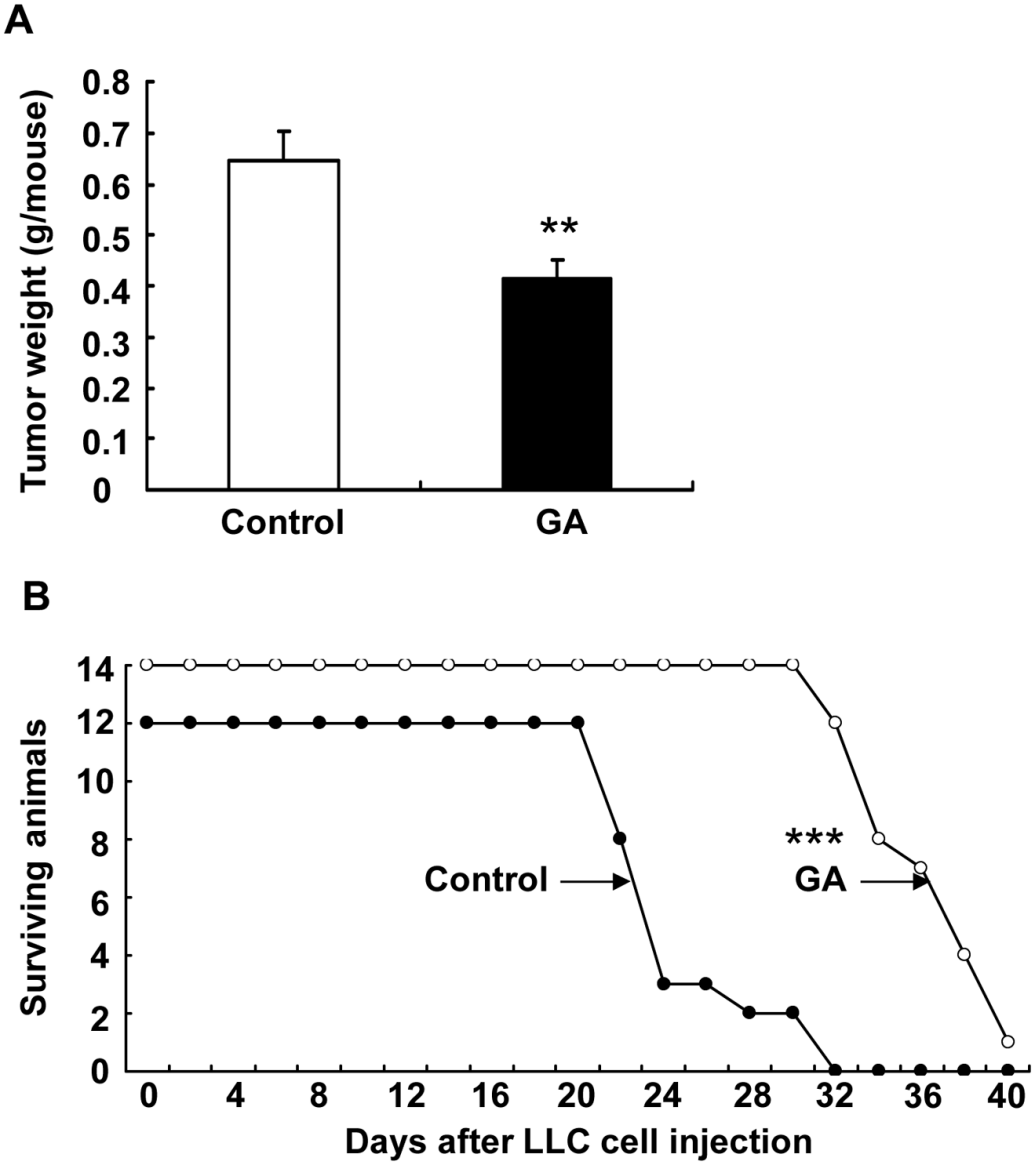
11βHSD2 inhibition suppressed lung tumorigenesis. **A**. LLC tumor growth was significantly attenuated by 11βHSD2 inhibition with GA (**P < 0.01, n = 8 in each group). LLC cell suspensions (100 μl, 5 x 10^5^ cells) were injected subcutaneously into the flank of C57/B6 mouse (2 sites). The mice were sacrificed 18 days later and tumor growth (tumor weight from two sites) was evaluated. **B**. Kaplan-Meier survival curve indicated that 11βHSD2 inhibition with GA increased survival probability in mice with tail vein injections of LLC cells (100 μl of LLC cell suspensions containing 5 x 10^5^ cells). ***P < 0.001, n = 12 in control and n = 14 in GA group. In both models, GA was given at 10 mg/kg/day (i.p. injection) starting one day before LLC cell injections.

To determine whether 11ßHSD2 inhibition affected lung tumorigenesis, we examined LLC tumor growth in a Xenograft model. LLC cells were injected subcutaneously into C57BL/6 mice. GA treatment (10 mg/kg/day, i.p.) was initiated one day before LLC cell injection and tumors were harvested and weighted at the end of the experiment (18 days after injection). As shown in Fig 4A, LLC tumor growth was markedly attenuated by GA treatment (tumor weight: 414 ± 38 vs. 646 ± 58 mg of vehicle, P **<** 0.01, n = 8 in each group). Similarly, GA treatment inhibited tumor invasion to the lung in a LLC tail vein injection model. As indicated in Fig 4B, GA treatment led to marked increase in survival probability (average survival days: 34.8 ± 3.3 vs. 24.5 ± 3.5 of control, P **<** 0.001, n = 12 in vehicle group and n = 13 in GA group).

**Fig 5:**
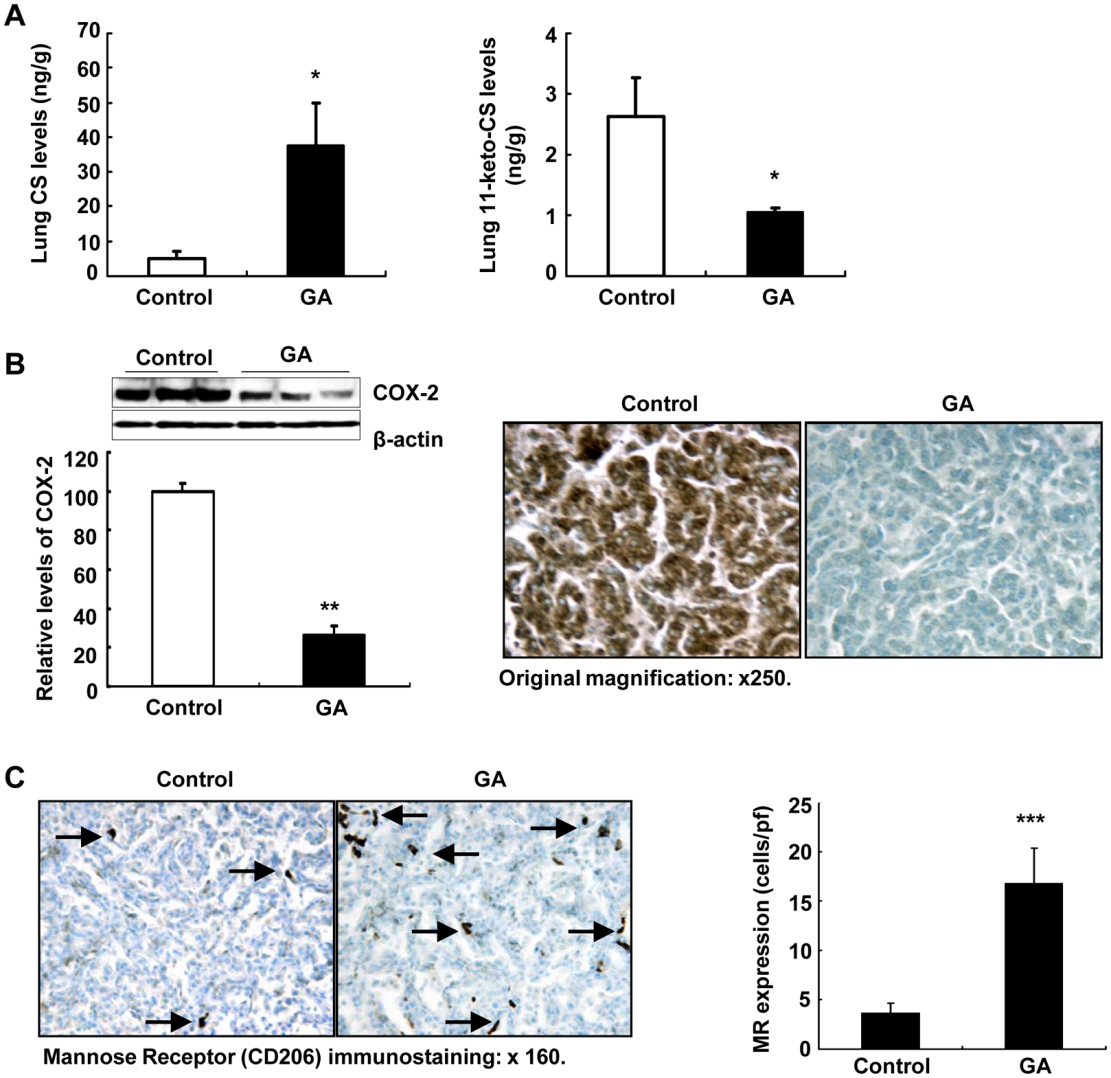
11βHSD2 inhibition with GA increased lung corticosterone levels in association with attenuation of tumor COX-2 expression in KrasLA2 mice. **A**. GA treatment markedly increased lung active corticosterone levels and decreased inactive 11-keto-corticosterone levels in KrasLA2 mice (20 weeks of age). *P <0.05 vs. control, n = 6. **B**. Lung tumor COX-2 expression was suppressed by 11ßHSD2 inhibition with GA. **P < 0.01 vs. control, n = 3. Immunostaining showed markedly decreased tumor COX-2 with GA treatment. Original magnification: x 250. **C**. GA treatment increased mannose receptor (MR, CD206) expressing macrophages in tumor, an indication of increased tumor levels of active corticosterone. ***P < 0.001 vs. control, n = 4. Original magnification: x 160.

KrasLA2 mice carry an oncogenic mutation of the *Kras* gene (G12D) and develop spontaneous primary tumors with features of NSCLC [24]. To further investigate the role of 11ßHSD2 inhibition in lung tumorigenesis, heterozygous KrasLA2 mice were treated with water (control) or GA (10 mg/kg/day, i.p.) from 6 weeks of age. At 20 weeks of age, a subset of mice was sacrificed, lung tissue kept for measurement of glucocorticoid levels and immunoblotting. Another subset of mice was sacrificed and the lungs were filled with fixative to fix the tissue for analysis of tumor number, size and immunostaining. Lung tissue levels of glucocorticoids were measured by using high-performance liquid chromatography coupled with electrospray tandem mass spectrometry [22]. GA treatment led to marked increases in lung corticosterone levels (active glucocorticoid in rodent: 37.40 ± 12.50 vs. 5.06 ± 2.19 ng/g lung tissue of control, P **<** 0.05, n = 6 in each group), and led to marked decreases in 11-keto-corticosterone (inactive glucocorticoid in rodent: 1.05 ± 0.08 vs. 2.63 ± 0.64 ng/g lung tissue of control, P **<** 0.05) (Fig 5A). As expected, COX-2 levels were markedly reduced in lung tumors from GA treated KrasLA2 mice (26 ± 5% of control, P < 0.001, n = 3 in each group) (Fig 5B). Immunostaining confirmed the reduction of tumor COX-2 expression in GA treatment mice. Glucocorticoids have been reported to induce mannose receptor (MR) expression in macrophages [30]. Therefore, we investigated MR expression in the tumor. As indicated in Fig 5C, the number of tumor MR-expressing cells was markedly higher in GA treated tumors than in control tumors (16.77 ± 3.58 vs. 3.64 ± 0.96 cells/pf, P < 0.001, n = 4 in each group).

**Fig 6:**
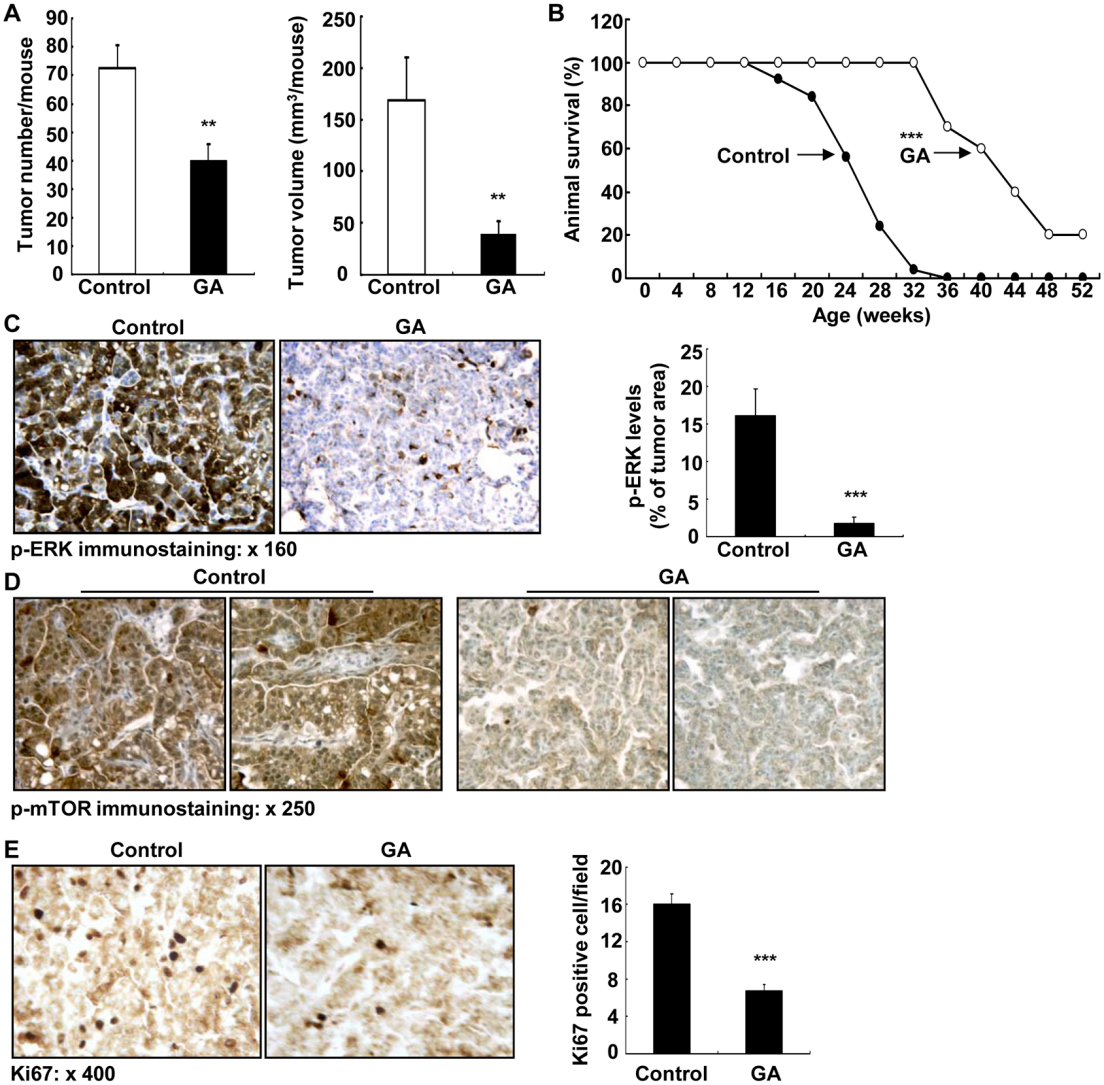
11βHSD2 inhibition suppressed lung tumorigenesis in association with suppression of tumor ERK and mTOR activities in KrasLA2 mice. **A**. GA treatment led to decreases in both number and size of lesion in lung surface of KrasLA2 mice (20 weeks of age). n = 9, **P < 0.01. **B**. Kaplan-Meier survival curve indicated that 11βHSD2 inhibition with GA increased survival probability of KrasLA2 mice. ***P < 0.001, n = 25 in vehicle group and n = 10 in GA group. **C**: GA treatment inhibited lung tumor levels of p-ERK. ***P < 0.001 vs. control, n = 4. Original magnification: x 160. **D**. GA treatment inhibited tumor p-mTOR expression. Original magnification: x 250. **E**. GA treatment inhibited tumor proliferation as indicated by decreased Ki67 positive cells in tumors. ***P < 0.001 vs. control, n = 4. Original magnification: x 400.

GA treatment caused significant inhibition of tumorigenesis in KrasLA2 mice, including decrease in adenoma number on the lung surface (40.0 ± 5.8 vs. 72.7 ± 7.8 of control, P **<** 0.01, n = 9 in each group) and decrease in adenoma size (mm^3^/mouse: 40.7 ± 10.7 vs. 168.8 ± 41 of vehicle, P **<** 0.01) (Fig 6A). GA treatment markedly increased lifespan of KrasLA2 mice (weeks: 42.1 ± 2.6 vs. 24.1 ± 1.0 of control, P **<** 0.01, n = 10 in GA group and n = 25 in control group) (Fig 6B). Therefore, GA treatment inhibited lung tumorigenesis in KrasLA2 mice and increased KrasLA2 mouse lifespan.

Glucocorticoids have been reported to inhibit lung cancer cell growth through inhibition of ERK activity [31,32]. Therefore, we investigated tumor ERK activity with immunostaining of phosphorylated ERK. As indicated in Fig 6C, the levels of tumor phosphorylated ERK were marked reduced by GA treatment (ratio of p-ERK area/tumor area: 1.79 ± 0.79 vs. 16.11 ± 3.61 of control, P < 0.001, n = 4 in each group). GA treatment also inhibited tumor mammalian target of rapamycin (mTOR) signaling pathway (Fig 6D). Finally, tumor cell proliferation was inhibited by GA treatment as indicated by decreased tumor cells with Ki67 positivity, a marker of cell proliferation (Fig 6E).

**Fig 7:**
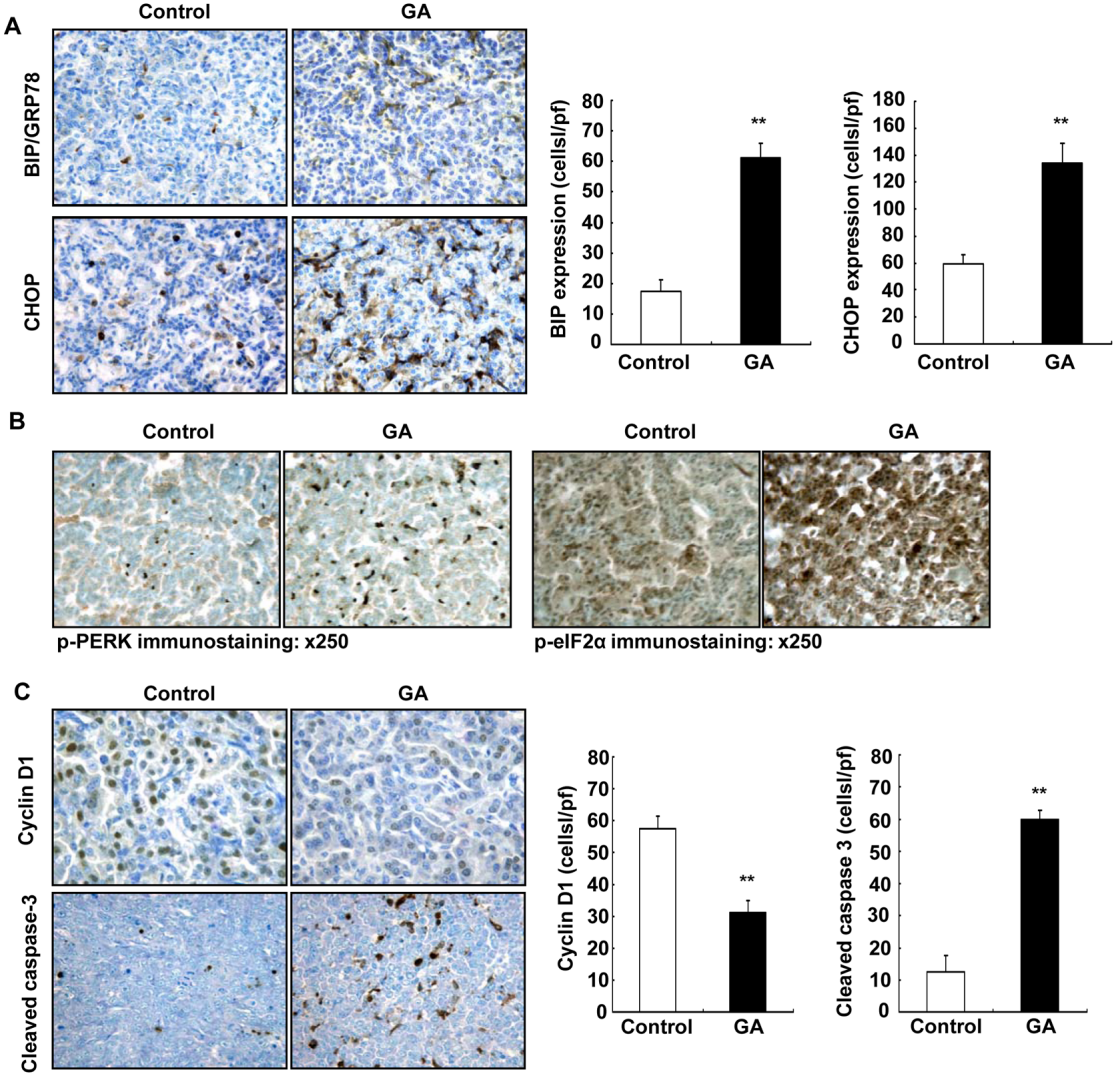
11ßHSD2 inhibition increased tumor endoplasmic reticulum (ER) stress in association with decreased cell proliferation and increased apoptosis in KrasLA2 mice. **A**. 11ßHSD2 inhibition led to increased expression of tumor BIP/GPR78 and CHOP, markers of ER stress. (**P < 0.01 vs. control, n = 4). Original magnification: x 160. **B**. Immunohistochemical staining indicated that tumor phosphorylated PERK and phosphorylated eIF2α levels were increased in mice with GA treatment. Original magnification: x 250. **C**. Immunostaining demonstrated that cyclin D1 (a marker of proliferation) was primarily localized to tumor cell nuclei, and its expression was markedly decreased in GA treated KrasLA2 mice (**P < 0.01 vs. control, n = 4). GA treatment increased tumor cell apoptosis as indicated by increase in cleaved-caspase-3 positive cells, a specific marker of apoptosis in KrasLA2 mouse lung tumors (**P < 0.01 vs. control, n = 4). Original magnification: x 250.

Recently, it was recognized that induction of endoplasmic reticulum stress (ER stress) and activation of the unfolded protein response (UPR) is an important pathway for antitumor agents to induce cancer cell death [33–35]. ER-stress has been reported to contribute to apoptosis induced by COX-2 inhibition in lung cancer cells as well as other cancer cells [36,37]. Furthermore, ER stress-mediated apoptosis requires eIF2α and PERK. Upon activation, PERK phosphorylates eIF2α to reduce global mRNA translation [38]. Therefore, we investigated the potential role of ER stress in 11ßHSD2 inhibition-induced suppression of lung tumorigenesis. C/EBP homologous protein (CHOP) plays an important role in ER stress induced apoptosis [36,37]. 11ßHSD2 inhibition with GA led to marked increases in BIP/GPR78 (a marker of ER stress) and CHOP expression in tumors from KrasLA2 mice (**P **<** 0.01, n = 4) (Fig 7A). In addition, 11ßHSD2 inhibition with GA led to increased phosphorylation of both PERK and eIF2α (Fig 7B).

To determine whether suppression of lung tumorigenesis by 11ßHSD2 inhibition was related to decreased cell proliferation and/or increased apoptosis, the expression levels of cyclin D1 (marker of proliferation) and cleaved caspase-3 (a specific marker of apoptosis) were investigated in KrasLA2 mouse tumors. GA treatment caused significant decreases in both cyclin D1 immunostaining density and the number of cyclin D1 positive cells in KrasLA2 lung tumors (cells/hpf: 31.1 ± 3.8 vs. 57.4 ± 4.0 of control, P **<** 0.01, n = 4). In contrast, the number of tumor cells that were positive for cleaved caspase-3 was significantly increased in GA-treated KrasLA2 lung tumors (cells/hpf: 59.8 ± 2.9 vs. 12.5 ± 5.2 of control, P **<** 0.01, n = 4) (Fig 7C). Therefore, inhibition of 11ßHSD2 activity with GA led to decreased cell proliferation and increased apoptosis in lung tumors.

## Discussion

The major findings in the present studies include: 1) 11ßHSD2 is expressed in epithelial cells of small airways and alveoli of mouse lung and its expression is increased in experimental lung tumors and in human SCLC and NSCLC, 2) glucocorticoid-induced COX-2 inhibition in lung cancer cells is enhanced by 11ßHSD2 inhibition, 3) inhibition of 11ßHSD2 activity with GA reduces LLC tumor growth and invasion, and 4) 11ßHSD2 inhibition with GA suppresses lung tumorigenesis and increases lifespan in KrasLA2 mice in association with increases in lung active corticosterone levels, decreases in tumor COX-2 expression, inhibition of the tumor ERK and mTOR signaling pathways and increased ER stress.

There is a clear link between activation of the COX-2/mPGES-1/PGE_2_ pathway and lung tumorigenesis and progression. In NSCLC A549 cells, COX-2-derived PGE_2_ promotes cell migration, cell proliferation and apoptosis resistance, and COX-2 inhibition suppresses cell proliferation associating with inhibition of survivin expression and increased caspase-3 mediated apoptosis [39–41]. The terminal enzyme of COX-2–mediated PGE_2_ biosynthesis, mPGES-1, plays a key role in LLC cell proliferation *in vitro* and tumor growth and metastasis *in vivo* [42]. COX-2 expression increased in experimental lung cancer models and COX-2 inhibition suppressed tumor development and growth in these models [43,44]. Selective COX-2 inhibition may act synergistically with ionizing radiation to inhibit A549 cancer cells through the activation of caspase-8 and caspase-3 [45]. Knockdown of mPGES-1 markedly reduced Xenograft A549 tumor growth [46].

GCs are known to inhibit cell proliferation and induce cell differentiation through activation of glucocorticoid receptors. 11ßHSD2 has been thought to be pro-proliferative due to its ability to inactivate glucocorticoids [47–50]. We have recently reported that 11ßHSD2 expression is increased in epithelial cells and stromal cells in human colonic and *Apc* ^*+/min*^ mouse intestinal adenomas and is correlated with increased COX-2 expression and activity, and that inhibition of 11ßHSD2 activity genetically or pharmacologically suppresses tumor COX-2 pathway and prevents adenoma formation, tumor growth, and metastasis as a result of increased tumor intracellular active glucocorticoids [22,51]. Sustained activation of the ERK signaling is important for lung cancer cell survival and proliferation [52,53]. GCs have been reported to inhibit lung cancer cell growth through inhibition of the ERK signaling pathway. Indeed, GA treatment led to inhibition of the tumor ERK signaling pathway in KrasLA2 mice (Fig 5C).

**Fig 8:**
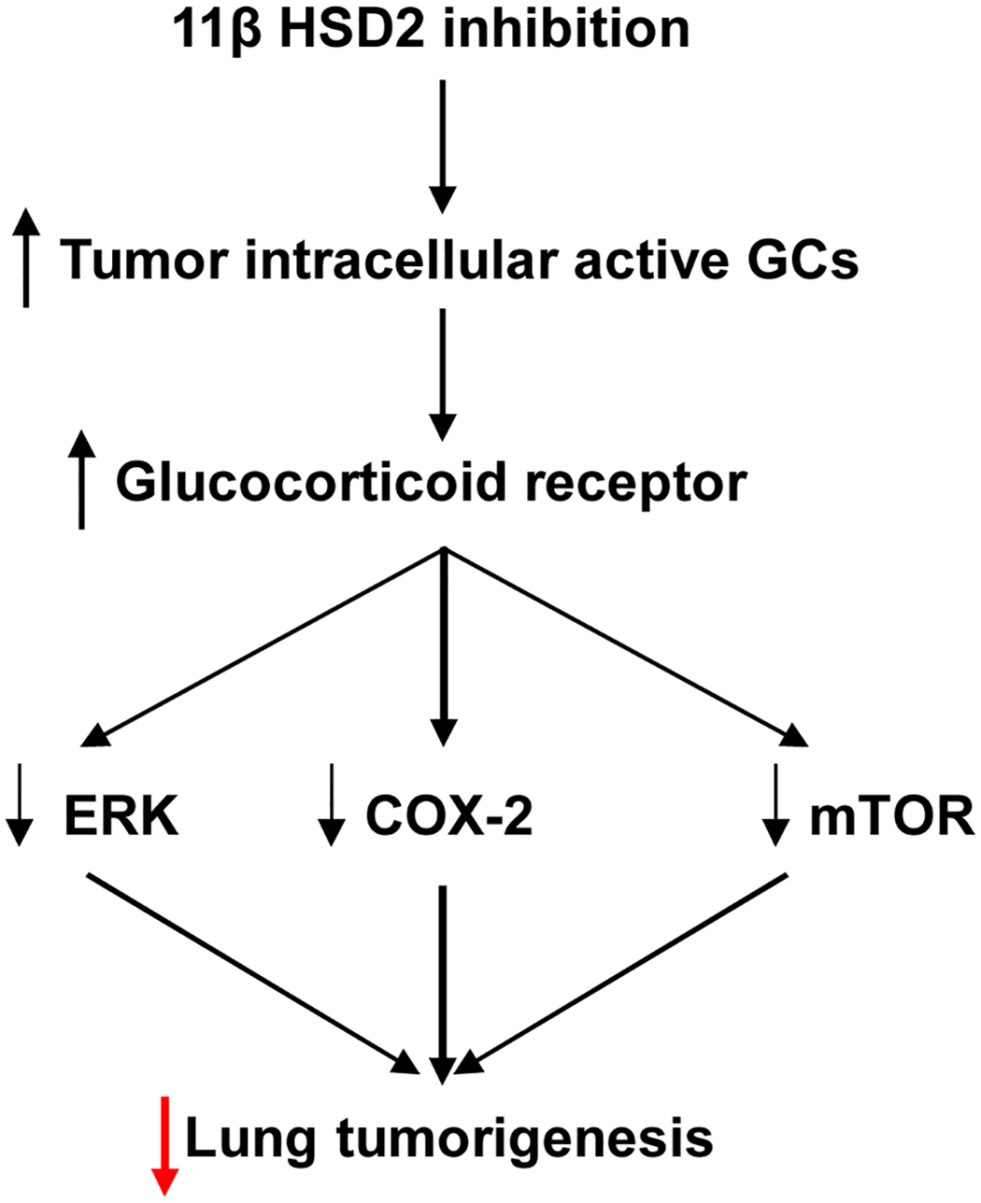
Proposed mechanism underlying 11ßHSD2 activity and lung tumorigenesis. 11ßHSD2 inhibition leads to increased levels of tumor intracellular active glucocorticoid and activation of glucocorticoid receptors. The subsequent inhibition of the COX-2, ERK and mTOR pathways leads to suppression of lung tumorigenesis.

Oncogenic K-Ras regulates proliferation and cell functions in lung epithelial cells through induction of cyclooxygenase-2 and activation of metalloproteinase-9 [54]. In the current study, inhibition of 11ßHSD2 activity led to inhibition of lung tumorigenesis in KrasLA2 mice associated with COX-2 inhibition and increased ER stress as well as inhibition of the ERK and mTOR signaling pathways due to increased tumor cell active glucocorticoids (Fig 8). Therefore inhibition of 11ßHSD2 activity with glycyrrhizic acid and its analogs may represent a novel approach for lung cancer chemoprevention, particularly in long-term heavy cigarette smokers, with the following advantages [22]: 1) GA, a natural compound contained in licorice, is a nontoxic, inexpensive and powerful 11ßHSD2 inhibitor; 2) Physiologic 11ßHSD2 expression is largely restricted to colon and kidney and lung. Therefore, inhibition of 11ßHSD2 activity is not expected to incur the cardiovascular risk posed by COX-2 inhibitors that suppress COX-2-derived PGI_2_ production in vascular endothelial cells; 3) Intracellular active GCs are only increased in tissues with elevated 11ßHSD2 expression. 11ßHSD2 inhibition will not produce immunosuppression or other systemic side effects of conventional glucocorticoid therapy; 4) In addition to inhibiting the COX-2 pathway, increased tumor active GCs also inhibit lung tumorigenesis through inhibiting the ERK and mTOR signaling pathways as well as induction of G1 cell cycle arrest [51]. Although 11ßHSD2 inhibition may result in salt-sensitive hypertension due to activation of mineralocorticoid receptors by GCs, development of locally acting 11ßHSD2 inhibitors that are not systemically absorbed would be a potential therapeutic means to prevent lung tumorigenesis [55]. In the future, it is worthy investigating whether MEK inhibitor or rapamycin can enhance GA-mediated anti-proliferative effect.

## References

[1] Houghton AM (2013) Mechanistic links between COPD and lung cancer. Nat Rev Cancer. 13: 233–245. 10.1038/nrc3477 23467302

[2] Kwon MC, Berns A (2013) Mouse models for lung cancer. Mol Oncol 7: 165–177. 10.1016/j.molonc.2013.02.010 23481268 PMC5528410

[3] Harris RC, Zhang MZ (2011) Cyclooxygenase metabolites in the kidney. Compr Physiol. 1: 1729–1758. 10.1002/cphy.c100077 23733687

[4] Wang D, Dubois RN (2010) Eicosanoids and cancer. Nat Rev Cancer. 10: 181–193. 10.1038/nrc2809 20168319 PMC2898136

[5] Hida T, Yatabe Y, Achiwa H, Muramatsu H, Kozaki K, Nakamura S, et al (1998) Increased expression of cyclooxygenase 2 occurs frequently in human lung cancers, specifically in adenocarcinomas. Cancer Res. 58: 3761–3764. 9731479

[6] Ho MY, Liang SM, Hung SW, Liang CM. (2013) MIG-7 controls COX-2/PGE2-mediated lung cancer metastasis. Cancer Res. 73: 439–449. 10.1158/0008-5472.CAN-12-2220 23149922

[7] Khuri FR, Wu H, Lee JJ, Kemp BL, Lotan R, Lippman SM, et al (2001) Cyclooxygenase-2 overexpression is a marker of poor prognosis in stage I non-small cell lung cancer. Clin Cancer Res. 7: 861–867. 11309334

[8] Bi N, Yang M, Zhang L, Chen X, Ji W, Ou G, et al (2010) Cyclooxygenase-2 genetic variants are associated with survival in unresectable locally advanced non-small cell lung cancer. Clin Cancer Res. 16: 2383–2390. 10.1158/1078-0432.CCR-09-2793 20332326

[9] Mao JT, Roth MD, Fishbein MC, Aberle DR, Zhang ZF, Rao JY, et al (2011) Lung cancer chemoprevention with celecoxib in former smokers. Cancer Prev Res (Phila). 4: 984–993. 10.1158/1940-6207.CAPR-11-0078 21733822 PMC3153413

[10] Komaki R, Liao Z, Milas L (2004) Improvement strategies for molecular targeting: Cyclooxygenase-2 inhibitors as radiosensitizers for non-small cell lung cancer. Semin Oncol. 31: 47–53. 14981580 10.1053/j.seminoncol.2003.12.014

[11] Bresalier RS, Sandler RS, Quan H, Bolognese JA, Oxenius B, Horgan K, et al (2005) Cardiovascular events associated with rofecoxib in a colorectal adenoma chemoprevention trial. N Engl J Med. 352: 1092–1102. 15713943 10.1056/NEJMoa050493

[12] Solomon SD, McMurray JJ, Pfeffer MA, Wittes J, Fowler R, Finn P, et al (2005) Cardiovascular risk associated with celecoxib in a clinical trial for colorectal adenoma prevention. N Engl J Med. 352: 1071–1080. 15713944 10.1056/NEJMoa050405

[13] Cheng Y, Austin SC, Rocca B, Koller BH, Coffman TM, Grosser T, et al (2002) Role of prostacyclin in the cardiovascular response to thromboxane A2. Science. 296: 539–541. 11964481 10.1126/science.1068711

[14] Clark AR, Lasa M. (2003) Crosstalk between glucocorticoids and mitogen-activated protein kinase signalling pathways. Curr Opin Pharmacol. 3: 404–411. 12901950 10.1016/s1471-4892(03)00073-0

[15] Newton R (2000) Molecular mechanisms of glucocorticoid action: what is important? Thorax. 55: 603–613. 10856322 10.1136/thorax.55.7.603 PMC1745805

[16] Zhang MZ, Harris RC, McKanna JA (1999) Regulation of cyclooxygenase-2 (COX-2) in rat renal cortex by adrenal glucocorticoids and mineralocorticoids. Proc Natl Acad Sci U S A. 96: 15280–15285. 10611376 10.1073/pnas.96.26.15280 PMC24811

[17] Croxtall JD, Gilroy DW, Solito E, Choudhury Q, Ward BJ, Buckingham JC, et al (2003) Attenuation of glucocorticoid functions in an Anx-A1-/- cell line. Biochem J. 371: 927–935. 12553880 10.1042/BJ20021856 PMC1223334

[18] Stichtenoth DO, Thoren S, Bian H, Peters-Golden M, Jakobsson PJ, Crofford LJ (2001) Microsomal prostaglandin E synthase is regulated by proinflammatory cytokines and glucocorticoids in primary rheumatoid synovial cells. J Immunol. 167: 469–474. 11418684 10.4049/jimmunol.167.1.469

[19] Denis MG, Chadeneau C, Blanchardie P, Lustenberger P (1992) Biological effects of glucocorticoid hormones on two rat colon adenocarcinoma cell lines. J Steroid Biochem Mol Biol. 41: 739–745. 1562548 10.1016/0960-0760(92)90415-f

[20] Schiffelers RM, Metselaar JM, Fens MH, Janssen AP, Molema G, Storm G (2005) Liposome-encapsulated prednisolone phosphate inhibits growth of established tumors in mice. Neoplasia. 7: 118–127. 15802017 10.1593/neo.04340 PMC1501128

[21] Funder JW, Pearce PT, Smith R, Smith AI (1988) Mineralocorticoid action: target tissue specificity is enzyme, not receptor, mediated. Science. 242: 583–585. 2845584 10.1126/science.2845584

[22] Zhang MZ, Xu J, Yao B, Yin H, Cai Q, Shrubsole MJ, et al (2009) Inhibition of 11beta-hydroxysteroid dehydrogenase type II selectively blocks the tumor COX-2 pathway and suppresses colon carcinogenesis in mice and humans. J Clin Invest. 119: 876–885. 10.1172/JCI37398 19307727 PMC2662561

[23] Yao B, Harris RC, Zhang MZ. (2005) Interactions between 11beta-hydroxysteroid dehydrogenase and COX-2 in kidney. Am J Physiol Regul Integr Comp Physiol. 288: R1767–1773. 15718388 10.1152/ajpregu.00786.2004

[24] Johnson L, Mercer K, Greenbaum D, Bronson RT, Crowley D, Tuveson FA, et al (2001) Somatic activation of the K-ras oncogene causes early onset lung cancer in mice. Nature. 410: 1111–1116. 11323676 10.1038/35074129

[25] Yang L, Yamagata N, Yadav R, Brandon S, Courtney RL, Morrow JD, et al (2003) Cancer-associated immunodeficiency and dendritic cell abnormalities mediated by the prostaglandin EP2 receptor. J Clin Invest. 111: 727–735. 12618527 10.1172/JCI16492 PMC151895

[26] Chang J, Jiang L, Wang Y, Yao B, Yang S, Zhang B, et al (2015) 12/15 lipoxygenase regulation of colorectal tumorigenesis is determined by the relative tumor levels of its metabolite 12-HETE and 13-HODE in animal models. Oncotarget. 6: 2879–2888. 25576922 10.18632/oncotarget.2994 PMC4413624

[27] Zhang MZ, Yao B, Cheng HF, Wang SW, Inagami T, Harris RCl (2006) Renal cortical cyclooxygenase 2 expression is differentially regulated by angiotensin II AT(1) and AT(2) receptors. Proc Natl Acad Sci U S A. 103: 16045–16050. 17043228 10.1073/pnas.0602176103 PMC1635124

[28] Harris RC, Zhang MZ, Cheng HF (2004) Cyclooxygenase-2 and the renal renin-angiotensin system. Acta Physiol Scand. 181: 543–547. 15283769 10.1111/j.1365-201X.2004.01329.x

[29] Sandeep TC, Yau JL, MacLullich AM, Noble J, Deary IJ, Walker BR, et al (2004) 11Beta-hydroxysteroid dehydrogenase inhibition improves cognitive function in healthy elderly men and type 2 diabetics. Proc Natl Acad Sci U S A. 101: 6734–6739. 15071189 10.1073/pnas.0306996101 PMC404114

[30] Schmieder A, Schledzewski K, Michel J, Schonhaar K, Morias Y, Bosschaets T, et al (2012) The CD20 homolog Ms4a8a integrates pro- and anti-inflammatory signals in novel M2-like macrophages and is expressed in parasite infection. European Journal of Immunology. 42: 2971–2982. 10.1002/eji.201142331 22806454

[31] Morita M, Suyama H, Igishi T, Shigeoka Y, Kodani M, Hashimoto K, et al (2007) Dexamethasone inhibits paclitaxel-induced cytotoxic activity through retinoblastoma protein dephosphorylation in non-small cell lung cancer cells. Int J Oncol. 30: 187–192. 17143528

[32] Greenberg AK, Hu J, Basu S, Hay J, Reibman J, Yie TA, et al (2002) Glucocorticoids inhibit lung cancer cell growth through both the extracellular signal-related kinase pathway and cell cycle regulators. Am J Respir Cell Mol Biol. 27: 320–328. 12204894 10.1165/rcmb.4710

[33] Jin HR, Zhao J, Zhang Z, Liao Y, Wang CZ, Huang WH, et al (2012) The antitumor natural compound falcarindiol promotes cancer cell death by inducing endoplasmic reticulum stress. Cell. Death Dis 3: e376 10.1038/cddis.2012.122 22914324 PMC3434669

[34] Um HJ, Bae JH, Park JW, Suh H, Jeong NY, Yoo YH, et al (2010) Differential effects of resveratrol and novel resveratrol derivative, HS-1793, on endoplasmic reticulum stress-mediated apoptosis and Akt inactivation. Int J Oncol. 36: 1007–1013. 20198347 10.3892/ijo_00000581

[35] Jakobsen CH, Storvold GL, Bremseth H, Follestad T, Sand K, Mack M, et al (2008) DHA induces ER stress and growth arrest in human colon cancer cells: associations with cholesterol and calcium homeostasis. J Lipid Res. 49: 2089–2100. 10.1194/jlr.M700389-JLR200 18566476 PMC2533412

[36] Kim BM, Maeng K, Lee KH, Hong SH (2011) Combined treatment with the Cox-2 inhibitor niflumic acid and PPARgamma ligand ciglitazone induces ER stress/caspase-8-mediated apoptosis in human lung cancer cells. Cancer Lett. 300: 134–144. 10.1016/j.canlet.2010.09.014 21067863

[37] White MC, Johnson GG, Zhang W, Hobrath JV, Piazza GA, Grimaldi M (2013) Sulindac sulfide inhibits sarcoendoplasmic reticulum Ca2+ ATPase, induces endoplasmic reticulum stress response, and exerts toxicity in glioma cells: relevant similarities to and important differences from celecoxib. J Neurosci Res. 91: 393–406. 10.1002/jnr.23169 23280445 PMC3595008

[38] Teske BF, Wek SA, Bunpo P, Cundiff JK, McClintick JN, Anthony TG, et al (2011) The eIF2 kinase PERK and the integrated stress response facilitate activation of ATF6 during endoplasmic reticulum stress. Mol Biol Cell. 22: 4390–4405. 10.1091/mbc.E11-06-0510 21917591 PMC3216664

[39] Qiu R, Chen J, Sima J, Shen X, Liu D, Shen J (2012) NS398 induces apoptosis in non-small cell lung cancer cells. J Cancer Res Clin Oncol. 138: 119–124. 10.1007/s00432-011-1080-3 22048655 PMC11824803

[40] Krysan K, Dalwadi H, Sharma S, Pold M, Dubinett S. (2004) Cyclooxygenase 2-dependent expression of survivin is critical for apoptosis resistance in non-small cell lung cancer. Cancer Res. 64: 6359–6362. 15374938 10.1158/0008-5472.CAN-04-1681

[41] Kim JI, Lakshmikanthan V, Frilot N, Daaka Y (2010) Prostaglandin E2 promotes lung cancer cell migration via EP4-betaArrestin1-c-Src signalsome. Mol Cancer Res. 8: 569–577. 10.1158/1541-7786.MCR-09-0511 20353998 PMC2855782

[42] Kamei D, Murakami M, Sasaki Y, Nakatani Y, Majima M, Ishikawa T, et al (2009) Microsomal prostaglandin E synthase-1 in both cancer cells and hosts contributes to tumour growth, invasion and metastasis. Biochem J. 425: 361–371. 10.1042/BJ20090045 19845504 PMC2825730

[43] Setia S, Vaish V, Sanyal SN (2012) Chemopreventive effects of NSAIDs as inhibitors of cyclooxygenase-2 and inducers of apoptosis in experimental lung carcinogenesis. Mol Cell Biochem. 366: 89–99. 10.1007/s11010-012-1286-y 22411738

[44] Rioux N, Castonguay A (1998) Prevention of NNK-induced lung tumorigenesis in A/J mice by acetylsalicylic acid and NS-398. Cancer Res. 58: 5354–5360. 9850065

[45] Kim BM, Won J, Maeng KA, Han YS, Yun YS, Hong SH (2009) Nimesulide, a selective COX-2 inhibitor, acts synergistically with ionizing radiation against A549 human lung cancer cells through the activation of caspase-8 and caspase-3. Int J Oncol. 34: 1467–1473. 19360361

[46] Hanaka H, Pawelzik SC, Johnsen JI, Rakonjac M, Terawaki K, Rasmuson A, et al (2009) Microsomal prostaglandin E synthase 1 determines tumor growth in vivo of prostate and lung cancer cells. Proc Natl Acad Sci U S A. 106: 18757–18762. 10.1073/pnas.0910218106 19846775 PMC2768589

[47] Rabbitt EH, Gittoes NJ, Stewart PM, Hewison M (2003) 11beta-hydroxysteroid dehydrogenases, cell proliferation and malignancy. J Steroid Biochem Mol Biol. 85: 415–421. 12943730 10.1016/s0960-0760(03)00224-3

[48] Hundertmark S, Buhler H, Rudolf M, Weitzel HK, Ragosch V (1997) Inhibition of 11 beta-hydroxysteroid dehydrogenase activity enhances the antiproliferative effect of glucocorticosteroids on MCF-7 and ZR-75-1 breast cancer cells. J Endocrinol. 155: 171–180. 9390020 10.1677/joe.0.1550171

[49] Lipka C, Mankertz J, Fromm M, Lubbert H, Buhler H, Kuhn W, et al (2004) Impairment of the antiproliferative effect of glucocorticosteroids by 11beta-hydroxysteroid dehydrogenase type 2 overexpression in MCF-7 breast-cancer cells. Horm Metab Res. 36: 437–444. 15305225 10.1055/s-2004-825724

[50] Koyama K, Myles K, Smith R, Krozowski Z. (2001) Expression of the 11beta-hydroxysteroid dehydrogenase type II enzyme in breast tumors and modulation of activity and cell growth in PMC42 cells. J Steroid Biochem Mol Biol. 76: 153–159. 11384873 10.1016/s0960-0760(00)00157-6

[51] Jiang L, Yang S, Yin H, Fan X, Wang S, Yao B, et al (2013) Epithelial 11beta-hydroxysteroid dehydrogenase type II deletion inhibits Apc+/min mouse tumorigenesis via COX-2 pathway inhibition and induction of G1 cell cycle arrest. Mol Cancer Res.

[52] Britson JS, Barton F, Balko JM, Black EP (2009) Deregulation of DUSP activity in EGFR-mutant lung cancer cell lines contributes to sustained ERK1/2 signaling. Biochem Biophys Res Commun. 390: 849–854. 10.1016/j.bbrc.2009.10.061 19836351

[53] Brognard J, Dennis PA (2002) Variable apoptotic response of NSCLC cells to inhibition of the MEK/ERK pathway by small molecules or dominant negative mutants. Cell Death Differ. 9: 893–904. 12181740 10.1038/sj.cdd.4401054

[54] Wang XQ, Li H, Van Putten V, Winn RA, Heasley LE, Nemenoff RA (2009) Oncogenic K-Ras regulates proliferation and cell junctions in lung epithelial cells through induction of cyclooxygenase-2 and activation of metalloproteinase-9. Mol Biol of the Cell. 20: 791–800. 10.1091/mbc.E08-07-0732 19037103 PMC2633382

[55] Stewart PM, Prescott SM (2009) Can licorice lick colon cancer? J Clin Invest. 119: 760–763. 19348044 10.1172/JCI38936 PMC2662579

